# Analysis of the TAX1BP1 gene in head and neck cancer patients

**DOI:** 10.1590/S1808-86942010000200008

**Published:** 2015-10-19

**Authors:** Mariangela Torreglosa Ruiz, Janayna Fernanda Balachi, Raquel Aldrighi Fernandes, Ana Lívia Silva Galbiatti, José Victor Maniglia, Érika Cristina Pavarino-Bertelli, Eny Maria Goloni-Bertollo

**Affiliations:** PhD in Health Sciences. Biologist - Genetics and Molecular Biology Unit - Medical School - São José do Rio Preto - FAMERP; Bachelor in Biology - UNESP São José do Rio Preto, Biologist; Medical Student - Medical School - São José do Rio Preto - FAMERP; Bachelor in Biology - UNORP - São José do Rio Preto, MSc in Health Sciences - Medical School - São José do Rio Preto; Associate Professor - Otolaryngology and Head and Neck Surgery, Adjunct Professor - Otorhinolaryngology and Head and Neck Surgery; Associate Professor in Human Genetics; MD; Adjunct Professor - Molecular Biology Department - Medical School -São José do Rio Preto - FAMERP; Associate Professor in Human Genetics; MD. Adjunct Professor - Medical School - São José do Rio Preto - FAMERP

**Keywords:** alcohol drinking, genetics, head and neck neoplasms, tobacco

## Abstract

In Brazil, there were 14,160 new estimated cases of head and neck cancer for the year of 2008. Smoking and drinking are the main risk factors established in the etiology of this disease.

**Aim:**

to assess the T → A polymorphism in gene TAX1BP1 (leu306ile) in patients with head and neck cancer and a control population.

**Series and methods:**

a retrospective study in which we assessed the gender, age, smoking and drinking habits of 191 patients with head and neck cancer and 200 individuals without history of neoplasia. The molecular analysis was carried out after genomic DNA extraction by the PCR-RFLP method.

**Results:**

there is a predominance of males (84.82%), smokers (91.1%) and drinkers of alcohol (77.49%). Molecular assessment did not show statistically significant differences between the two groups (p =0.32). The analysis of clinical parameters and polymorphisms showed association with oral cavity cancer (OR: 2.38; CI 95%: 1.18-4.78; p = 0.01), the other parameters were not associated with the polymorphism.

**Conclusion:**

There is evidence of association between TAX1BP1 gene polymorphism and oral cavity cancer. For the remaining parameters analyzed, the results do not suggest association with the TAX1BP1 gene polymorphism.

## INTRODUCTION

Malignant neoplasia of the head and neck is a term associated with tumoral lesions located in the oral cavity (40%), pharynx (15%), larynx (25%), and other places -such as the salivary glands (20%)^1^^,^^2^. The most frequent histological type is the squamous cell carcinoma, present in more than 90% of the cases^3^^,^^4^. This type of cancer is characterized by a major local aggressiveness, recurrence. About 2/3 of the patients have the disease in advanced stages, commonly involving the regional lymph nodes. Distant metastasis happened to 10% of the patients^5^^,^^6^.

In Brazil, according to the National Cancer Institute (NCI), the incidence of oral cancer - the most representative among these types of tumor, was estimated in 14,160 new cases in 2008^7^.

Smoking and drinking are the main risk factors associated with head and neck cancer. Epidemiological studies have shown that each one of these factors separately increases the risk of developing neoplasia in this region in three fold, and when associated, the risk goes up to be fifteen fold^8^, ^9^, ^10^.

Single nucleotide polymorphisms (SNPs) are the most common type of variation in the human genome and correspond to DNA polymorphisms affecting one single nucleotide, with a frequency higher than 1% in humans^11^, ^12^, ^13^, ^14^. An ever larger number of these alterations is being associated to the molecular bases of diseases involving a genetic component, such as cancer, or as a risk factor for acquired diseases^14^.

Numerous haplotypes and genetic polymorphisms involved in the metabolism of drugs, transportation and action mechanisms have been investigated in order to optimize treatment response, such as C677T and A1298C polymorphisms of the methylenetetrahydrofolate reductase (MTHFR)gene^15^.

In this study, we assessed the role of a single nucleotide polymorphism T → A, in the development of head and neck carcinoma, present in gene TAX1BP1, described by Brentani et al. in 2003^16^. This gene is a marker of interest for the study for having been selected in an unbiased library (Cancer Human Genome Project), which passed through a first validation stage^17^.

Gene TAX1BP1 produces Human T-cell leukemia virus type I binding protein 1, and is located in chromosome 7p15 and has a T → A polymorphism resulting in the substitution of Leucine (leu) for Isoleucine (ile) in position 306 of the protein18. It was identified for the first time as a target of the Tax protein from virus of the human T cell leukemia^20^. TAX1BP1 also interacts with other important molecules such as A20 and TRAF6, which participate in inflammatory response processes^19^^,^^20^.

Thus, the goal of the present paper was to identify the T → A polymorphism, which results in the substitution of aminoacid Leucine (leu) for Isoleucine (ile) in position 306 of the protein from gene TAX1BP1 in patients with head and neck cancer and in a population of individuals without a past of neoplasia and also to assess the distribution of these genotypes according with the clinical and pathological characteristics of head and neck cancer.

## MATERIALS AND METHODS

This study was approved by the Ethics in Research Committee of the institution under protocol # 5566/2005.

In this paper we analyzed 191 patients with head and neck cancer and 200 individuals without a past of neoplasia. All the samples were obtained after free and informed consent from all the participants. We analyzed the social and demographic profile of these patients (age and gender) and the exposure to risk factors (smoking and drinking). Information about smoking and drinking was limited regarding the use or not of smoke and alcohol beverages. Those individuals who smoked about 100 cigarettes during their entire lives were considered smokers and those who had more than four drinks per week were considered alcoholics^21^^,^^22^. In the group of patients we included those individuals with a histopathological diagnosis of head and neck squamous cell carcinoma. In the control group we included individuals from outpatient wards of other medical specialties, as exclusion criteria we investigated the individual's cancer family history, ruling out the cases of neoplasias. We used 60 months as follow up time for controls and patients.

Among patients, we analyzed the primary sites of tumor occurrence and the tumors were classified according to the parameters of the Union International Control Cancer (IUCC), 2002 and the American Joint Committee for Cancer (AJCC), 2002, in three criteria: tumor size (T), compromised regional lymph nodes present (N) and the presence of distant metastases (M)^23^^,^^24^.

Genomic DNA was extracted from peripheral blood^25^. For molecular evaluation, we used the PCR-RFLP method. In order to identify the polymorphic alleles we used polymerase chain reactions (PCR) in a total volume of 20 μl, using 50 ng of genomic DNA, 0.3mM of sense primer (5′ - ACCTGGGTCTCCTAAATCCT – 3′), 0.3 mM of antisense primer (5′ - AGCCTG CCAATCTCTTCTT – 3′), 200mM dNTP, 1X reaction buffer, 1.5mM MgCl2, 0.2 units of Taq polymerase. The PCR amplification was carried out through an initial denaturation stage at 95°C, followed by 35 cycles of denaturation at 95°C for 30 seconds, annealing at 57°C, extension at 72°C for 30 seconds and final extension stage at 72°C. The PCR products were visualized in 1.5% agarose gel, dyed with ethidium bromide.

The amplification products were submitted to enzyme digestion with Apo I restriction endonuclease and this digestion product was submitted to a 2% agarose gel electrophoresis at 80 V for 4 hours in order to observe the migration pattern among the size fragments. Polymorphism destroys the restriction site, and the amplified 264 pb is not cut. In the absence of polymorphism, the product is digested in two fragments (one fragment of 205 pb and another fragment of 59 pb).

For the statistical analysis, the demographic data was plotted by descriptive analysis and compared through the Fisher's Exact Test. This test was also used for the statistical analysis of the genotypic and allelic polymorphism distribution.

The multiple logistic regression models were used in order to determine the effect of the variables analyzed in head and neck cancer. The model included age (reference: < 52 years, median of the two groups), gender (reference: female), smoking (reference: non-smokers) and drinking habits (reference: not a drinker).

The clinical-pathological characteristics were also analyzed by multiple logistic regressions. T classification was broken down in small tumors (T1, T2) and larger ones (T3, T4The N classification was dichotomized into negative lymph node involvement (N0), and positive (N1, N2, N3). Stages were broken down into early (stages I, II) and advanced disease (III and IV).

The results were presented in odds ratio (OR) and 95% confidence interval (CI – 95%). The level of significance was established in 5% (p=0.05).

The Kaplan-Meier method was employed in order to assess different disease survival and recurrence rates among the different genotypes and the Log-Rank test was used in order to assess these differences between genotypes. For the survival analysis we considered as end point the period comprehended between disease diagnosis and death, and for the recurrence analysis, the end point was the recurrence diagnosis.

## RESULTS

Table 1 shows the demographic data of the 191 patients with head and neck cancer and the 200 individuals without a past of neoplasias. The mean age of patients with head and neck cancer was 58 years, with a standard deviation of ± 9.69. We used a control group (individuals without a history of neoplasia) with mean age of 47 years, with a standard deviation of ± 15.69. The statistical analysis for this variable was significant between the groups (p< 0.0001). The median age of patients and controls was 42 years.

**Table 1:** Demographics of head and neck cancer patients.

	Patients	Controls	P
# of individuals	191	200	
Age (mean ±SD)	58.24±9.69	47.55±15.69	< 0.0001
Gender			0.04
Males	162 (84.82%)	153 (76.5%)	
Females	29 (15.18%)	47(23.5%)	
Smoking	174 (91.1%)	102 (51%)	< 0.0001
Drinking	148 (77.49%)	102 (51%)	< 0.0001

SD= Standard Deviation

As far as gender is concerned, 84.82% of the patients were males and 15.18% were females. The frequency found in the control group was 76.5% of males and 23.5% females. The statistical analysis showed significance between these two groups (p= 0.04).

In relation to smoking and drinking habits, the information was limited to whether or not the person smoked or drank alcohol. In the group of patients with head and neck cancer, 91.1% were smokers and 77.49% drank alcoholic beverages. Among control individuals, 51% were smokers and 51% drank alcoholic beverages. The statistical analysis concerning smoking proved significant among the groups (p<0.0001), as well as alcohol consumption (p<0.0001). The most representative tumor site among patients was the oral cavity (30%).

The allele frequencies for the head and neck cancer patients were, respectively (T=0.87 and A=0.13) and for controls (T=0.90 and A=0.10) not showing statistically significant difference between the groups (p=0.32). The genotypic frequency (Table 2) also did not show difference between the groups (p= 0.3080). The genotypic frequencies are within the Hardy-Weinberg balance between patients (X2 = 1.71; p = 0.19) and control groups (X2 = 0.05; p = 0.81).

**Table 2:** Genotypic distribution of the TAX1BP1 gene T→A polymorphism (Genotypes TT: wild homozygote; TA: heterozygote; AA: polymorphic homozygote) in head and neck cancer patients and controls.

Genotypes	Patients n (%)	Controls n (%)	p
TT	148 (82,41)	165 (82,41)	0,3080
TA	38 (19,90)	33 (16,58)	
AA	5 (2,62)	2 (1,01)	

Since it was not possible to pair the demographic data with risk factors between patients and controls, we carried out the multiple logistic regression in order to assess the potential interaction between the genotypes studied and the socio-demographic characteristics (age and gender) and risk factors (smoking and drinking) (Table 3) and there were no differences between the groups regarding the characteristics investigated.

**Table 3:** Demographic data distribution, risk factors, TAX1BP1 genotypes (genotypes TT: wild homozygote; TA: heterozygote; AA: polymorphic homozygote) in odds ratio (OR) for the head and neck cancer.

Variables	TT (Patients/Controls)	OR (CI 95%)*	TA and AA (Patients/Controls)	OR (CI 95% CI)	p
Gender					
Female	22/37	1,00 (ref)	07/10	0,95 (0,27-3,32)	0,94
Male	126/130	1,00 (ref)	36/23	1,37 (0,68–2,75)	0,37
Age**					
< 52	38/115	1,00 (ref)	14/25	1,08 (0,46- 2,51)	0,86
> 52	110/52	1,00 (ref)	29/08	1,30 (0,53- 3,16)	0,52
Cigarette smoking					
No	14/87	1,00 (ref)	03/11	2,57 (0,52–12,83)	0,25
Yes	134/80	1,00 (ref)	40/22	1,19 (0,63–2,24)	0,59
Alcohol drinking					
No	31/87	1,00 (ref)	11/11	2,23 (0,67 – 7,43)	0,19
Yes	32/80	1,00 (ref)	117/22	1,02 (0,51 −2,03)	0,96

* Adjusted for age, gender, cigarette smoking and alcoholic beverages drinking; OR = odds ratio; CI = confidence interval

** Group age median calculation

**Figure 1:**
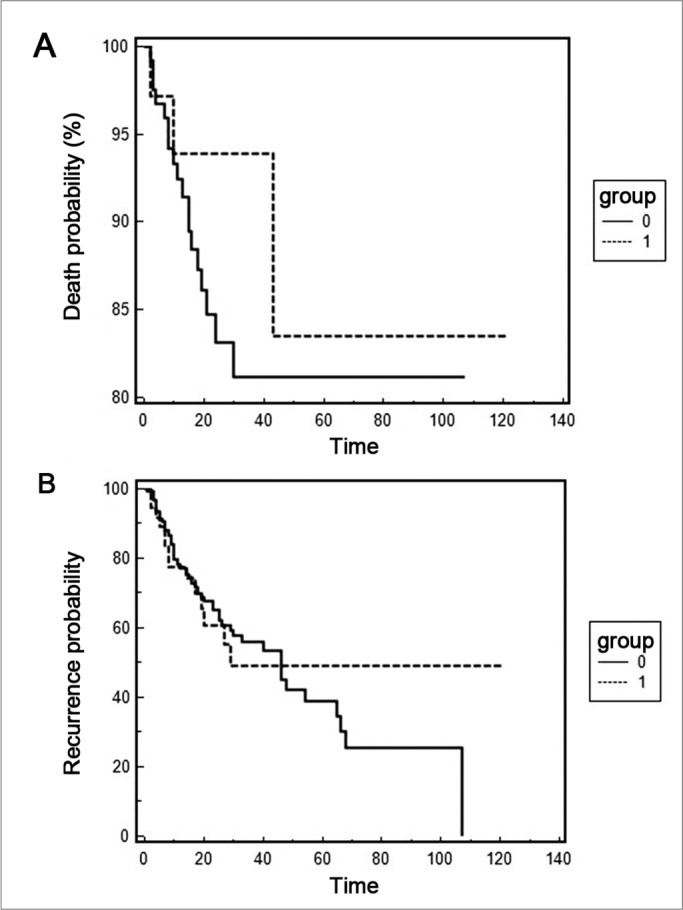
Kaplan-Meier curve for death time (A) and disease recurrence time (B). Group 0 represents the TT genotype and group 1 the TA and AA genotypes.

The analysis between the polymorphism and the clinical parameters is presented on Table 4. Regarding the tumor's primary anatomical site, the polymorphism frequency was greater in the oral cavity cancer (OR = 2.38; IC 95% 1.18 – 4.78; p = 0.01). We did not find associations regarding tumor extension, lymph node involvement and tumor staging. The analysis for the M criterion was not carried out since only one patient had the M1 classification. The Kaplan-Meier curves are presented on Figure 1, and there were no statistical differences between the presence of polymorphism, survival rate (p = 0.4078) and the disease recurrence rate (p = 0.8827). For this analysis we calculated the presence of recurrence and death after the first consultation (disease diagnosis) until the consultation date in the medical chart. Regarding recurrence, there was an average of 27 months and only 02 patients (2.11%) died during the analysis period.

**Table 4:** Clinical and pathological characteristics and TAX1BP1 polymorphism (Genotypes TT: wild homozygote; TA: heterozygote; AA: polymorphic homozygote)

Variables	TT genotype	OR (CI 95%)	TA and AA genotypes	OR (CI 95%)*	p
Tumor sites**					
Oral cavity	48	1,00 (ref)	23	2,38 (1,18 – 4,78)	0,01
Pharynx	48	1,00 (ref)	08	0,49 (0,21 – 1,15)	0,10
Larynx	48	1,00 (ref)	11	0,70 (0,32 – 1,52)	0,36
Tumor extension***					
T1/T2	46	1,00 (ref)	14	1,12 (0,53 – 2,38)	0,77
T3/T4	89	1,00 (ref)	25	1,09 (0,52 – 2,31)	0,81
Lymph node involvement***					
No	74	1,00 (ref)	24	1,00 (ref)	
Yes	67	1,00 (ref)	15	0,67 (0,32 – 1,40)	0,28
Stages***					
I/II	34	1,00 (ref)	11	1,00 (ref)	
III/IV	94	1,00 (ref)	26	0,82 (0,36- 1,87)	0,65

* *Adjusted for age, gender, cigarette smoking and alcoholic beverages drinking.

** Unknown primary anatomical sites were taken off.

*** Analysis carried out for patients with complete pathology data

## DISCUSSION

This study matched literature data regarding the epidemiology of head and neck cancer, which is more frequent in men after their fifth decades of life, smokers and those who drink alcoholic beverages. Epidemiologic evidence has shown that the incidence of head and neck cancer increases with age^2^^,^^3^.

Such disease is relatively rare in women; especially in developing countries, males are more affected than females^1^^,^^26^. Although this neoplasia preferably affects men, in recent years we have seen a notable increase in its incidence in women, which should reflect changes in smoking and drinking habbits^9^^,^^10^.

Regarding risk factors, smoking and drinking alcoholic beverages are the main factors involved in the development of head and neck neoplasia - according to the literature^8^, ^9^, ^10^ and this study showed this association.

The genotypic frequencies are within the Hardy-Weinberg balance.

The results did not show significant differences between the groups studied and the TAX1BP1 gene polymorphism, although there are studies correlating the presence of such gene and the neoplasm development, especially leukemias^18^^,^^27^. There are also gene expression studies showing increased TAX1BP1 gene expression in ovary cancer^28^ and rheumatoid arthritis^29^. It is known that the protein interacts with the A20 (anti-apoptotic activity). Such protein causes complex NF-KB activation, which increases proliferation, cell death protection and eventually cell transformation, although these mechanisms are not well understood^30^.

The literature does not show any description of the frequencies of these polymorphisms and their respective effects on head and neck cancer, and there are no Brazilian studies on this subject - which further highlights the importance of the present investigation.

The polymorphic allele frequency was greater in the oral cavity cancer and can be explained by the histological differences which are peculiar to the different anatomical regions. Other studies suggest that the biological behavior of this tumor type is different in different anatomic locations^31^.

Numerous studies analyzed the association of clinical and pathological characteristics of patients with head and neck cancer and polymorphisms. As to tumor aggressiveness, in this study we did not notice association between this polymorphism and extension (T category) and tumor staging in the three major head and neck regions analyzed (oral cavity, pharynx and larynx). The study by Teng et al. in 2009^32^, analyzed the relationship between SDF-1-30A and CXCR4 genes polymorphisms and showed a relation between the latter with advanced stage mouth tumors (III and IV). CYP1E1*5B and null GSTM1 polymorphisms were also associated to advanced stages of the disease^33^.

The association of these polymorphisms and head and neck cancer, as well as their association with responses to carcinogens can help understand the mechanisms involved in the neoplastic process and the setting up of strategies to prevent this disease.

## CONCLUSION

There is evidence of this association between the TAX1BP1 gene polymorphism and oral cavity cancer. There are no differences in the distribution of the polymorphism among patients with head and neck cancer and individuals without history of neoplasm. There was also no difference in genotype distribution between the different tumor sizes, lymph node involvement, staging, recurrence and death.
